# Neurological Research and Practice – the premier journal of the German Society of Neurology: recent development and future perspectives

**DOI:** 10.1186/s42466-025-00372-5

**Published:** 2025-02-14

**Authors:** Werner Hacke, Wolf-Rüdiger Schäbitz

**Affiliations:** 1 Universitätsklinik für Neurologie, Rupprecht-Karls Universität, Heidelberg, Germany; 2 Universitätsklinik für Neurologie, Evangelisches Klinikum Bethel, Universität Bielefeld, Bielefeld, Germany

Five years ago, on February 28, 2019 a series of 7 articles was released in the first edition of Neurological Research and Practice (NRP), the new open access online only journal of the German Society of Neurology (DGN), published in English by BMC, a subsidiary of Springer Nature. The aim of the German Society of Neurology was to make the dimensions and quality of neurological research and clinical practice in Germany better known in the international scientific community. Of course, German neurologists have been publishing quite successfully in leading international journals for decades and some German neuroscientists and clinicians are among the most renowned and cited researchers in the world, but we believe that this does not fully reflect the very active and innovative scientific base of German neurology. Now, after 5 years, the journal has achieved broad acceptance, is listed in all relevant index sites, and has received the first Impact Factor from Clavariate in 2024 and the first Citescore (Elsevier) in 2023.

With this editorial, we would like to highlight some important achievements, point out interesting details, and announce changes in the journal’s editorial board that will come into effect in 2025 and 2026, respectively.

## Metrics of the journal

After registering with PubMed, EMBASE, SCOPUS and the Web of Science, the journal received its first CiteScore as early as 2023, which was the earliest possible time given the algorithms behind this measurement. The first CiteScore issued in 2023 was 5.7 for 2022 and became 7.4 for 2023. In 2024, the first Journal Impact Factor of 3.6 was published for 2023. Both impact measures place the journal in the highest quintile of clinical neurology and neuroscience journals. The journal’s 5-year impact factor is 4.5 for 2023. The h5 index for 2022 is 23.

## Publications

**Fig. 1:**
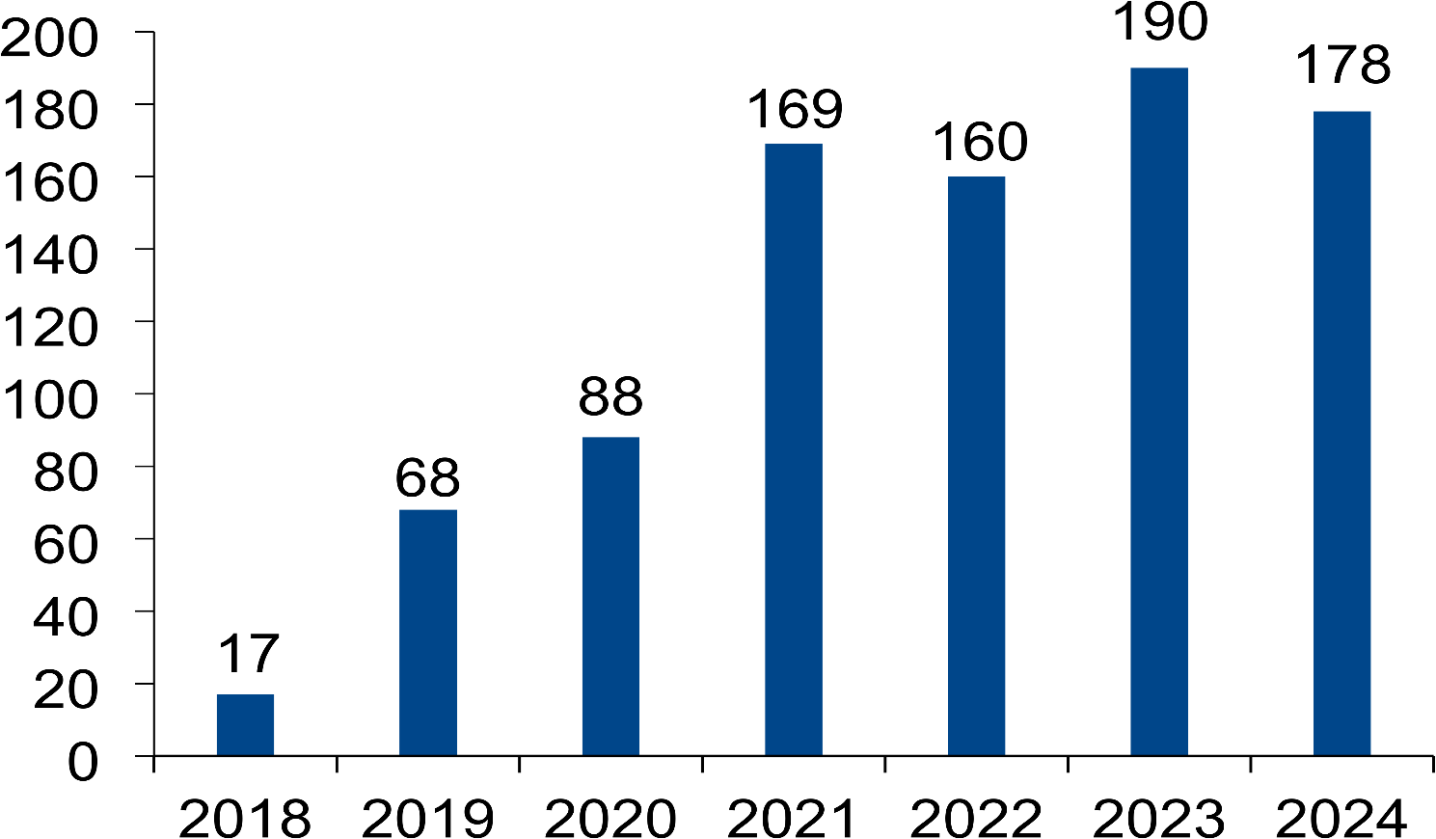
Submissions to Neurological Research and Practice between 2018 and 2024

**Fig. 2:**
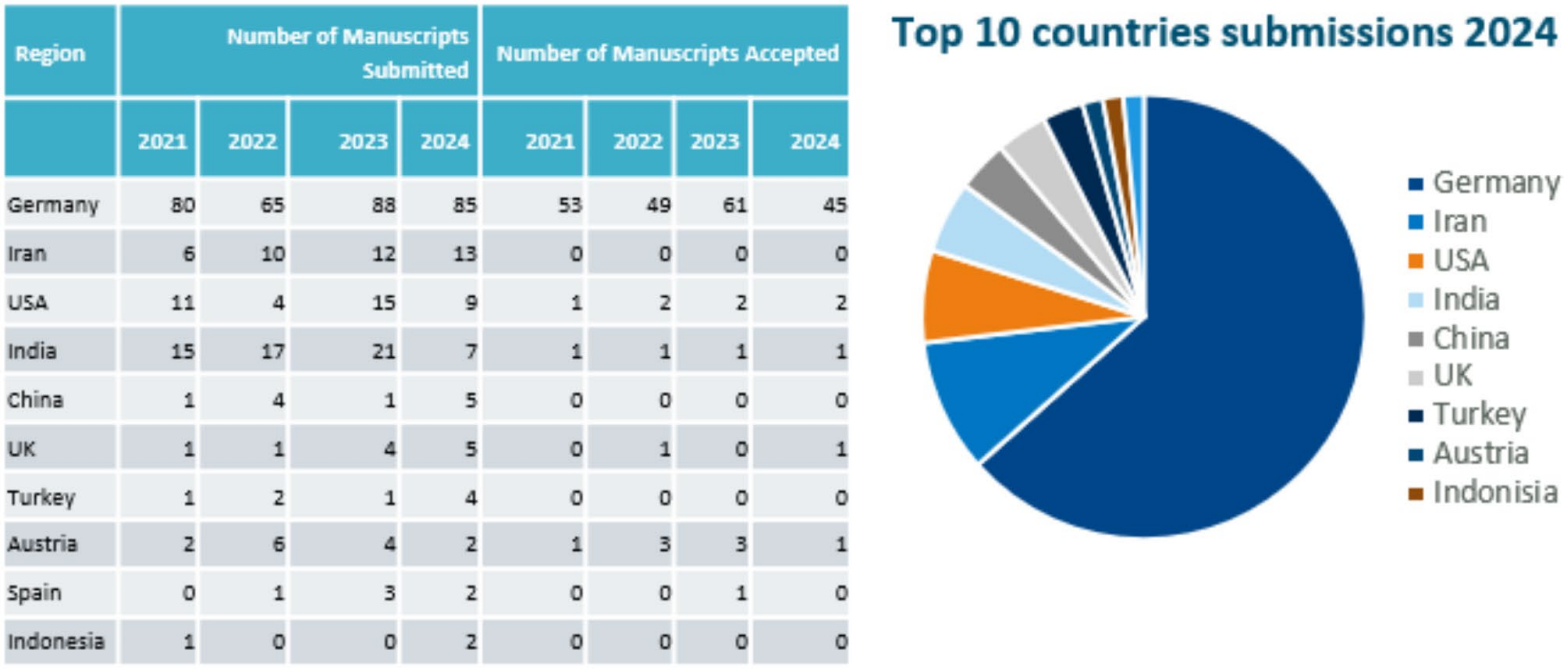
Author region of origin of submitted and accepted manuscripts

The journal publishes reviews, research reports, correspondence (formerly letters), selected abbreviated guidelines of the German Society of Neurology in English, clinical study protocols and standard operating procedures. The annual number of submissions is shown in Fig. 1. It has now stabilized at around 175 submissions per year. The rejection rate has reached 72%. While initially the majority of submissions came from Germany we have received more international than German submissions in the last two years (Fig. 2).

**Fig. 3:**
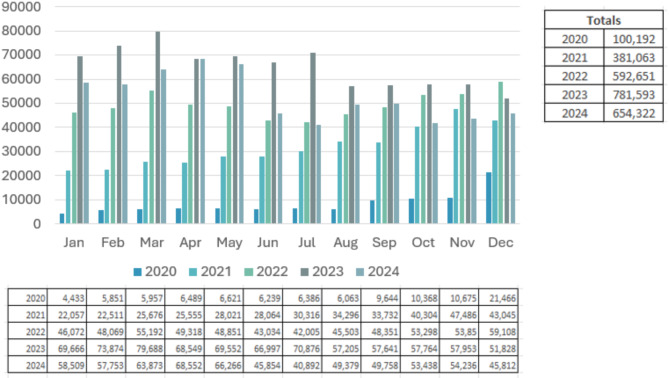
Full text article downloads between 2020–2024 according to COUNTER usage data on Google BigQuery. Downloads from SpringerLink, Nature.com and BMC Platform

Figure 3 shows the monthly successful downloads of full-text articles over a period of five years, which have now leveled at between 60.000 and 70.000 downloads per month. The leading countries for article downloads are the USA, Canada, the UK, the Philippines, Australia and India.

Table 1 lists the 10 most frequently accessed articles between 2019 and 2023. It is interesting to note that almost all publication types are represented in this list, even 2 letters are among the top 10 most accessed publications. Table 2 shows the list of the 10 most cited articles from the same period. Some articles made it into both lists. Importantly, SOP articles and clinical trial protocols are also included in this list.

**Table 1:** 10 most frequently accessed articles between 2019 and 2024

Title	Author	Article Type	Year	Total Article Requests
How to use and assess qualitative research methods	Loraine Busetto, Wolfgang Wick, Christoph Gumbinger	Review Paper	2020	838,000
Recovery from stroke: current concepts and future perspectives	Christian Grefkes, Gereon R. Fink	Review Paper	2020	89,000
Diagnosis of peripheral neuropathy	Helmar C. Lehmann, Gilbert Wunderlich, Gereon R. Fink, Claudia Sommer	Original Paper	2020	84,000
Increased incidence of transient global amnesia during the Covid-19 crisis?	Ralph Werner, Moritz Keller, Johannes C. Woehrle	Letter	2020	44,000
“Neurological manifestations of COVID-19” - guideline of the German society of neurology	Peter Berlit, Julian Bösel, Georg Gahn, Stefan Isenmann, Sven G. Meuth, Christian H. Nolte, Marc Pawlitzki, Felix Rosenow, Benedikt Schoser, Götz Thomalla, Thomas Hummel	Guidelines	2020	40,000
Diagnosis and treatment of neurogenic dysphagia – S1 guideline of the German Society of Neurology	Rainer Dziewas, Hans-Dieter Allescher, Ilia Aroyo, Gudrun Bartolome, Ulrike Beilenhoff, Jörg Bohlender, Helga Breitbach-Snowdon, Klemens Fheodoroff, Jörg Glahn, Hans-Jürgen Heppner, Karl Hörmann, Christian Ledl, Christoph Lücking, Peter Pokieser, Joerg C. Schefold, Heidrun Schröter-Morasch, Kathi Schweikert, Roland Sparing, Michaela Trapl-Grundschober, Claus Wallesch, Tobias Warnecke, Cornelius J. Werner, Johannes Weßling, Rainer Wirth, Christina Pflug	Original Paper	2021	41,000
Guideline “diagnosis and non interventional therapy of neuropathic pain” of the German Society of Neurology (deutsche Gesellschaft für Neurologie)	Tanja Schlereth	Letter	2020	26,000
Resumption of oral anticoagulation after spontaneous intracerebral hemorrhage	Jochen A. Sembill, Joji B. Kuramatsu, Stefan Schwab, Hagen B. Huttner	Review	2019	23,000
Glymphatic system, AQP4, and their implications in Alzheimer’s disease	Inês Silva, Jéssica Silva, Rita Ferreira, Diogo Trigo	Review	2021	20,000
Insomnia in neurological diseases	Geert Mayer, Svenja Happe, Stefan Evers, Wiebke Hermann, Sabine Jansen, Ulf Kallweit, Maria-Lucia Muntean, Dieter Pöhlau, Dieter Riemann, Michael Saletu, Melanie Schichl, Wolfgang J. Schmitt, Friederike Sixel-Döring, Peter Young	Guidelines	2021	19,000

**Table 2:** 10 most frequently cited articles between 2019 and 2024

Title	Author	Article Type	Year	Total Citations
How to use and assess qualitative research methods	Loraine Busetto, Wolfgang Wick, Christoph Gumbinger	Review Paper	2020	373
Recovery from stroke: current concepts and future perspectives	Christian Grefkes, Gereon R. Fink	Review Paper	2020	208
Glymphatic system, AQP4, and their implications in Alzheimer’s disease	Diogo Trigo, Inês Silva, Jéssica Silva, Rita Ferreira	Review	2021	105
Diagnosis of peripheral neuropathy	Helmar C. Lehmann, Gilbert Wunderlich, Gereon R. Fink, Claudia Sommer	Original Paper	2020	79
Safety and clinical impact of FEES - results of the FEES-registry	Rainer Dziewas, Matthias Auf Dem Brinke, Ulrich Birkmann, Götz Braeuer, Kolja Busch, Franziska Cerra, Renate Damm-Lunau, Juliane Dunkel, Amelie Fellgiebel, Elisabeth Garms, Jörg Glahn, Sandra Hagen, Sophie Held, Christine Helfer, Mirko Hiller, Christina Horn-Schenk, Christoph Kley, Nikolaus Lange, Sriramya Lapa, Christian Ledl, Beate Lindner-Pfleghar, Marion Mertl-Rötzer, Madeleine Müller, Hermann Neugebauer, Duygu Özsucu, Michael Ohms, Markus Perniß, Waltraud Pfeilschifter, Tanja Plass, Christian Roth, Robin Roukens, Tobias Schmidt-Wilcke, Beate Schumann, Julia Schwarze, Kathi Schweikert, Holger Stege, Dirk Theuerkauf, Randall S. Thomas, Ulrich Vahle, Nancy Voigt, Hermann Weber, Cornelius J. Werner, Rainer Wirth, Ingo Wittich, Hartwig Woldag, Tobias Warnecke	Research article	2019	77
“Neurological manifestations of COVID-19” - guideline of the German society of neurology	Peter Berlit, Julian Bösel, Georg Gahn, Stefan Isenmann, Sven G. Meuth, Christian H. Nolte, Marc Pawlitzki, Felix Rosenow, Benedikt Schoser, Götz Thomalla, Thomas Hummel	Guidelines	2020	59
Computer-aided imaging analysis in acute ischemic stroke - background and clinical applications	Simon Nagel, Yahia Mokli, Johannes Pfaff, Daniel Pinto dos Santos, Christian Herweh	Review	2019	53
Distribution and evolution of acute interventional ischemic stroke treatment in Germany from 2010 to 2016	Jens Eyding, Ralph Weber, Kitzrow, Martin; Dirk Bartig, Christian Weimar, Werner Hacke, Christos Krogias	Research article	2019	37
Neurological symptoms in COVID-19: a cross-sectional monocentric study of hospitalized patients	Ummehan Ermis, Marcus Immanuel Rust, Julia Bungenberg;; Ana Costa, Michael Dreher, Paul Balfanz, Gernot Marx, Martin Wiesmann, Kathrin Reetz, Simone C. Tauber, Jörg B. Schulz	Research article	2021	42
An omics-based strategy using coenzyme Q10 in patients with Parkinson’s disease: concept evaluation in a double-blind randomized placebo-controlled parallel group trial	Christine Klein, Jannik Prasuhn, Norbert Brueggemann, Nicole Hessler, Daniela Berg, Thomas Gasser, Kathrin Brockmann, Denise Olbrich, Andreas Ziegler, Inke R. König, Christine Klein, Meike Kasten	Clinical trial protocol	2019	35

## Organization of the journal

The German Society of Neurology had appointed Werner Hacke, Professor Emeritus of Neurology at the University of Heidelberg, to serve as the founding Editor-in-Chief of NRP. In the first two years, all manuscripts were edited exclusively by him, with significant support from an international editorial board. With the increase in submissions, we tested a system with more than 25 section editors assigned by the editor-in-chief and working as editing editors. The new structure was introduced in 2022 and worked reasonably well but caused some delays in processing manuscripts. From 2025, we will work with a smaller group of Associate Editors and Editorial Advisors for Statistics, History, Neuropediatrics, Neuroradiology and Genetics (further information on the Journal Webpage https://neurolrespract.biomedcentral.com).

The Editorial Board has also undergone major changes and now includes young professionals who were very productive and helpful in the early years of the journal. Most importantly, the current EiC will step down by the end of 2025 and the next EiC has already been appointed by the German Neurological Society: It will be Professor Wolf-Rüdiger Schäbitz, Professor and Chair of the Department of Neurology at Evangelisches Klinikum Bethel and Bielefeld University. His bioscetch has been added to the journal´s website starting page. In 2025, we will both share the tasks of the EiC to ensure a smooth transition. Our collaboration has already begun and has proven to be a smooth process.

I (WH) would like to thank the Presidents and the board members of the German Neurological Society for their trust in offering me the founding of this journal and for their continuous support during the first years. I am grateful for the help of the Managing Director of the Society and his team and for quick solutions when unexpected hurdles arose. I am more than grateful to all the active members of the editorial board and the section editors for their continuous support and perseverance when reviewers did not respond, did not deliver on time or declared unavailability after weeks. We all know how difficult it is to find reviewers who deliver high quality reviews - a big thank you to all of them. The most responsive and reliable colleagues have been recognized and have been promoted to the Editorial board. For details regarding the editorial board please visit the Journal Webpage https://neurolrespract.biomedcentral.com and open “Editorial Board”.

## Future development

As documented by publications, impact factor and cite score, as well as other journal metrics, Neurological Research and Practice has become a visible voice of the German Neurology Society. This achievement will be further expanded and developed. Modifications of the editorial board as presented above, further internationalization, the use of multimedia presentation and the augmentation of social media activities will be deployed to attract readers and authors thereby leading to a continuous influx of high profile original manuscripts, review articles and guidelines.

